# Elevated reactive aggression in forebrain‐specific *Ccn2* knockout mice

**DOI:** 10.1002/ccs3.12040

**Published:** 2024-07-20

**Authors:** Ho‐Ching Chang, Chi‐Hou Ng, Yu‐Fu Chen, Yu‐Chun Wang, I‐Shing Yu, Lukas Jyuhn‐Hsiarn Lee, Li‐Jen Lee, Kuang‐Yung Lee

**Affiliations:** ^1^ College of Medicine Graduate Institute of Anatomy and Cell Biology National Taiwan University Taipei Taiwan; ^2^ Department of Neurology Chang Gung Memorial Hospital Keelung Branch Keelung Taiwan; ^3^ Department of Otolaryngology, Head and Neck Surgery Chi‐Mei Medical Center Tainan Taiwan; ^4^ Laboratory Animal Center College of Medicine National Taiwan University Taipei Taiwan; ^5^ National Institute of Environmental Health Sciences National Health Research Institutes Miaoli Taiwan; ^6^ College of Medicine Institute of Brain and Mind Sciences National Taiwan University Taipei Taiwan; ^7^ Neurobiology and Cognitive Science Center National Taiwan University Taipei Taiwan; ^8^ College of Medicine Chang Gung University Taoyuan Taiwan

**Keywords:** c‐fos, medial amygdala, olfactory system, prefrontal cortex, resident‐intruder test

## Abstract

Cellular communication network factor 2 (CCN2) is a matricellular protein that plays important roles in connective tissue. CCN2 is also expressed in the nervous system; however, its role is still unclear. To explore CCN2 function in the brain, we generated forebrain‐specific *Ccn2* knockout (Fb*Ccn2* KO) mice. In this study, we examined the behavioral phenotypes of Fb*Ccn2*KO mice. Male mice lacking CCN2 in the forebrain exhibited normal locomotion, sensorimotor gating, and social behaviors but signs of anxiety and elevated reactive aggression. We checked the c‐fos expression in aggression‐related brain regions following the resident‐intruder task (RIT), an aggression test. RIT‐induced c‐fos levels in the medial amygdala (MeA) were higher in Fb*Ccn2* ^−/−^ mice as compared to controls. However, in the prefrontal cortex, RIT‐induced c‐fos levels in Fb*Ccn2* ^−/−^ mice were lower than controls. Our results suggested in male mice lacking CCN2 in the olfaction‐related regions, olfactory social cues elicit greater signals in the MeA, resulting in greater reactive aggression in the RIT. Further, lacking CCN2 in the prefrontal cortex, the major area related to inhibitory control and emotion regulation, may lead to signs of anxiety and the failure to suppress aggressive behaviors. Our model is useful in elaborating the mechanism underlying reactive aggression and therapeutic strategies.

## Graphical abstract

Cellular communication network factor 2 (CCN2) is expressed in the olfactory bulb, anterior olfactory nucleus, frontal cortex (FC), and cortical layer IVb. Intruder mice elicit greater neuronal signals in the medial amygdala, in mice lacking CCN2, resulting in greater reactive aggression during resident‐intruder task. Further, lacking CCN2 in the FC, may lead to the failure of suppressing aggressive behaviors.

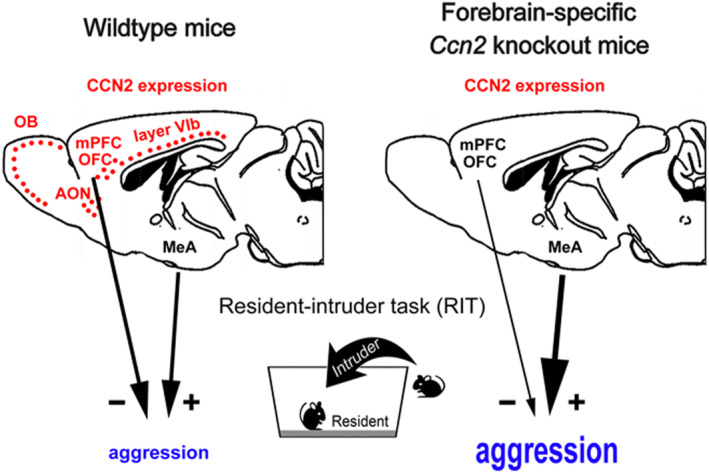

## 1 INTRODUCTION

Cellular communication network factor 2 (CCN2), also known as connective tissue growth factor (CTGF), is a cysteine‐rich secreted matricellular protein^1^ that plays important roles in the development and regeneration of the connective tissue in various organs.^2^, ^3^, ^4^, ^5^, ^6^, ^7^ Interestingly, CCN2 expression is not restricted to the connective tissue but also in the forebrain structures, such as the olfactory bulb (OB), anterior olfactory nucleus (AON), endopiriform nucleus (EPN), and cortical layer IVb,^8^, ^9^, ^10^, ^11^, ^12^, ^13^, ^14^ CCN2 expression in the brain has been reported using CCN2‐EGFP mice. Based on the dendritic morphology, the GFP signals are expressed in neurons.^15^ Besides, the expression of CCN2 has been reported in the nervous system under pathological or stressful conditions.^12^, ^16^, ^17^, ^18^, ^19^, ^20^, ^21^, ^22^, ^23^, ^24^, ^25^, ^26^ The roles of CCN2 in the nervous system are still not clear.

Among these CCN2‐expressing forebrain structures, the OB, AON, and EPN are involved in the olfactory function. In the OB, CCN2 is expressed in the glomerular and mitral cell layers,^11^ which contain the excitatory neurons that control output signals.^27^, ^28^ The projecting neurons in the OB send axon fibers to various brain regions, including the frontal cortex and amygdala (AMY), which are highly associated with the status of emotion and aggression.^29^, ^30^, ^31^ Blocking of olfactory inputs or removal of the OB, olfactory bulbectomy, is well known to substantially influence emotion, sociability, and aggression in rodents.^32^, ^33^, ^34^ Removal of *Ccn2* in the OB of neonatal mice by local viral injection affects the cytoarchitecture and function of OB neurons as well as the sensitivity to olfactory cues.^11^ We wonder if mice lacking *Ccn2* in the forebrain, especially the olfaction‐related regions, exhibit altered emotional and social behaviors.

In order to elucidate the function of CCN2 in the forebrain, we generated forebrain‐specific *Ccn2* conditional knockout (Fb*Ccn2* KO) mice by crossing *Ccn2* ^fl/fl^ mice with *Emx1‐Cre* mice.^13^ *Emx1* is a homeobox gene expressed in the excitatory neurons and some glial cells in the forebrain structures.^35^ In these KO mice, the full‐length *Ccn2* gene is removed by Cre‐mediated gene deletion and the CCN2 protein is undetectable in the forebrain while they are viable without apparent abnormality.^13^ We showed that CCN2 may influence cell morphology and physiology in the brain in an autocrine and paracrine manner.^13^ These mutant mice also exhibit delayed seizure response, reduced c‐fos expression, and different microglial phenotypes following acute PTZ injection.^14^

In the present study, we examined the expression pattern of CCN2 in the forebrain, characterized behavioral phenotypes of Fb*Ccn2* KO mice, and checked the c‐fos expression following the resident intruder test, an examination of aggressive behavior. Elevated reactive aggression and altered c‐fos expression patterns were noticed in these KO mice.

## 2 MATERIALS AND METHODS

### 2.1 Animals

Forebrain‐specific conditional *Ccn2* knockout (Fb*Ccn2* KO) mice were generated as previously described.^13^ In the present study, mating pairs of *Emx1‐Cre*; *Ccn2* ^fl/+^ mice were established, and their offspring, *Emx1‐Cre*; *Ccn2* ^+/+^ (Fb*Ccn2* ^+/+^), *Emx1‐Cre*; *Ccn2* ^fl/+^ (Fb*Ccn2* ^+/−^), and *Emx1‐Cre*; *Ccn2* ^fl/fl^ (Fb*Ccn2* ^−/−^), were used. Animals were bred and kept in the Laboratory Animal Center of the College of Medicine, National Taiwan University, under a 12‐h light/dark cycle (lights off at 08:00) with free access to food and water. Genotypes of the mice were examined using PCR‐based protocol, and the expression of CCN2 protein was checked by CCN2 immunohistochemistry.^13^ Adult (12–18 weeks of age) mice were used in this study. All animal procedures were approved by the Institutional Animal Care and Use Committee of the College of Medicine, National Taiwan University (approval code: 20170291).

### 2.2 Behavioral tests

The behavioral tests were carried out during the dark phase. Before tests, mice were placed in the experimental environment for habituation. The behaviors of mice were videotaped and examined using the TopScan software (CleverSys, Reston, VA, USA) by experimenters blinded to genotypes. Upon completion of each trial and test, the apparatus and the objects were carefully cleaned with 10% alcohol and dried.

#### 2.2.1 Open field test

The locomotor activity and anxiety state of mice were examined in the open field test. In brief, a mouse was placed in the open field apparatus (white acrylic square box of 45 × 45 × 50 cm in size) and allowed to move freely for 30 min. The spontaneous activities of a mouse were continuously recorded and subsequently traced.

#### 2.2.2 Prepulse inhibition (PPI) of the acoustic startle response

The sensorimotor gating property of mice was evaluated by the PPI test, as previously described.^36^ In brief, an individual mouse was placed in the cylinder of the startle chamber (SR‐LAB, San Diego Instrument, San Diego, CA, USA) and exposed to 65 dB background noise. Startle stimulus (120 dB) alone, no stimulus, and prepulse‐pulse pairs of 68–120 dB, 71–120 dB, or 77–120 dB were randomly given. The startle responses were recorded, and the PPI (%) was calculated.

#### 2.2.3 Forced swim test

Each mouse was confined in a Plexiglas cylinder (25 cm height, 10 cm internal diameter) filled with water (25°C, 15 cm height) for 6 min. Three behavioral parameters, including struggling (mouse stretched forepaws out of the water to escape), immobility (mouse remained calmly in the water with its head above the water and maintained balance for more than 2 s), and swimming (mouse tried to keep its body from sinking by moving its hind paws), were noted.

#### 2.2.4 Elevated plus maze

The elevated plus maze was used to evaluate the anxiety status of mice. The maze was constructed with white acrylic boards as previously described.^37^ During the test, a mouse was placed on the central platform and allowed to move freely for 10 min. The distance traveled and time spent in the open arms, closed arms, and central region were quantified.

#### 2.2.5 Three‐chamber social interaction test

The sociability of mice was evaluated using the three‐chamber test. A Plexiglas cage (35 × 30 × 96 cm) was divided into three equal regions (35 × 30 × 32 cm). Before the test, a mouse was allowed to explore the chambers for 10 min. We then put a target mouse in a small Plexiglas cylinder in one side chamber (social chamber), and an empty Plexiglas cylinder was placed in another side chamber (object chamber). Afterward, the test mouse was placed in the central chamber and allowed to explore freely for 10 min. The time spent in each chamber was recorded.

#### 2.2.6 Resident‐intruder task (RIT)

An adult male mouse of Fb*Ccn2* ^+/+^ and Fb*Ccn2* ^−/−^ was housed with an adult female mouse in their home cages for 10 days. On the test day, the female mouse was removed, and an adult male intruder with similar body weight was placed into the cage. In the 20 min of social contact, aggressive behavior indexes, including attack time, duration, and frequency, as well as latency to the first attack, were measured.

### 2.3 Histological examinations

Two hours after the exposure to the intruders, the resident mice were overdosed with 150 mg/kg sodium pentobarbital and transcardially perfused with 0.1 M PBS, followed by 4% paraformaldehyde. Whole brains were then harvested and postfixed overnight in the same fixative.

#### 2.3.1 Immunohistochemistry

Brain sections were cut and processed as previously described.^38^ In brief, sections were taken and incubated in the blocking solution containing 4% normal goat serum, 1% bovine serum albumin, and 0.4% TritonX‐100 in PBS. After 2 hours of blocking, the sections were incubated with primary antibodies, including goat anti‐CCN2 (1:1000; Santa Cruz Biotechnology, Santa Cruz, CA, USA), rabbit anti‐c‐fos (1:1000, Cell Signaling, Danvers, MA, USA), or anti‐NeuN (1:500; Merck Millipore, Darmstadt, Germany) in 10% blocking solution overnight. After washing, the sections were incubated with biotinylated secondary antibodies (1:500, Jackson ImmunoResearch Laboratories, West Grove, PA, USA) and avidin‐biotin‐peroxidase complex (ABC kit, Vector Labs, Burlingame, CA, USA). Finally, sections were reacted with 3, 3′‐diaminobenzidine (with 0.01% H_2_O_2_ in PBS) and mounted. For control experiments, we omitted the use of primary antibodies, and the immunoreactive signals were neglectable.

#### 2.3.2 Cell density quantification

The densities of c‐fos‐ and NeuN‐positive nuclei were quantified by measuring the number of cells within a counting frame (100 × 100 or 200 μm × 200 μm) in given brain regions using the ImageJ software (NIH, Bethesda, MD, USA).

### 2.4 Statistical analysis

Data were expressed as mean ± SEM. Statistical analyses were performed between different groups using two‐tailed unpaired student's *t*‐test or univariate analysis of variance. Asterisks were used to indicate significant differences (*p < 0.05; **p < 0.01; ***p < 0.001).

## 3 RESULTS

### 3.1 Absence of CCN2 expression in the brain of conditional knockout mice

**FIGURE 1:**
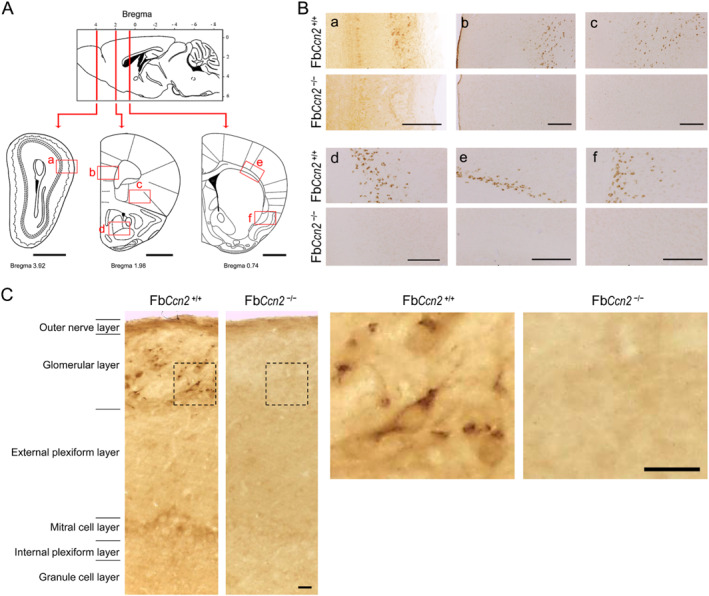
Expression of CCN2 in the mouse brain. Sections were collected from various coronal plans (A). CCN2 expression was revealed using immunohistochemistry (B, C). In Fb*Ccn2* ^+/+^ mice, CCN2‐positive cells were labeled in the glomerular layer of the OB (a), medial prefrontal cortex (b), orbitofrontal cortex (c), anterior olfactory nucleus (d), the cortical VIb (e) and the endopiriform nucleus (f). In higher magnitude images of the OB, CCN2‐positive signals were evident in the glomerular layer of the OB in Fb*Ccn2* ^+/+^ mice (C). In Fb*Ccn2* ^−/−^ mice, the expression of CCN2 was not detected (B, C). Scale bar = 1 mm in (A), 200 μm in (B), and 20 μm in (C). OB, olfactory bulb.

By crossing *Emx1‐Cre* mice with *Ccn2* ^fl/fl^ mice, forebrain‐specific *Ccn2* knockout (Fb*Ccn2* KO) mice were generated.^13^ We first examined the expression of CCN2 in the forebrain using immunohistochemistry (Figure 1). CCN2 protein was detected in the glomerular layer of the OB, (Fig. 1Ba and C). These cells are presumably external tufted cells.^11^ Besides, CCN2 protein was also detected in the deep portion of the medial prefrontal cortex (mPFC; Fig. 1Bb), orbitofrontal cortex (OFC; Fig. 1Bc), AON (Fig. 1Bd), the cortical layer VIb (Fig. 1Be) and the endopiriform nucleus (EPN; Fig. 1Bf). Based on the morphology of immunostained cells, we believe that CCN2 is expressed in neurons although the possibility of glial expression cannot be excluded. Nevertheless, the expression of CCN2 was absent in Fb*Ccn2* ^−/−^ mice (Figure 1B,C).

### 3.2 Signs of anxiety in Fb*Ccn2* ^−/−^ male mice

**FIGURE 2:**
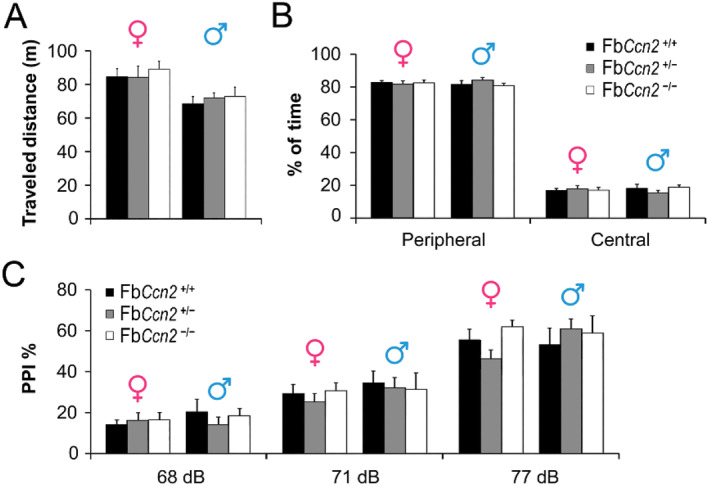
Locomotor activity and sensorimotor gating property of mice. The traveled distance of mice in an open field was measured. In both genders, no difference was noticed among genotypes (A). The time spent in the peripheral and central regions of the open field was also measured. In both sexes, no difference was noted among the three genotypes (B). The sensorimotor gating property was evaluated by the acoustic startle response. The ratios of prepulse inhibition (PPI) following different prepulse stimuli (68 dB, 71 dB, and 77 dB) were comparable among genotypes in both sexes (C). *N* = 5 mice in each condition. Results are mean ± SEM.

Since there is no significant difference in external appearance among Fb*Ccn2* ^+/+^, Fb*Ccn2* ^+/−^, and Fb*Ccn2* ^−/−^,^13^ we evaluated the behavioral phenotypes of these mice. The locomotor activities of mice were examined in the open field test. Both male and female mice were placed in the open field; the traveled distance and the time spent in the central and peripheral regions were comparable among genotypes (Figure 2A,B). Given that the locomotor activity is not affected by forebrain *Ccn2* removal, we then examined the sensorimotor gating property of these mutant mice. The ratio of prepulse inhibition (PPI) of the acoustic startle response was measured. No difference was noted among genotypes (Figure 2C).

**FIGURE 3:**
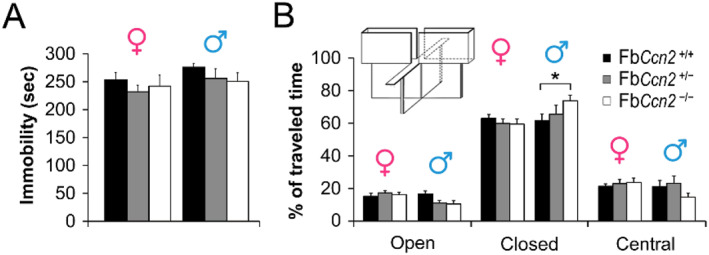
Depression and anxiety level of mice. The forced swim test was used to evaluate the level of depression. In both genders, the duration of immobility was comparable among genotypes (A). The anxiety level of mice was assessed using an elevated plus maze. The time spent in open arms, closed arms, and the central region was measured (B). Male Fb*Ccn2* ^−/−^ mice spent more time in closed arms than male Fb*Ccn2* ^+/+^ mice, indicating a high anxiety level. In female mice, the time spent in all regions was comparable among genotypes. *N* = 5 mice in each condition. Results are mean ± SEM. *p < 0.05.

We next elucidated the emotional states in Fb*Ccn2* KO mice. Both male and female mice were subjected to the forced swim test to study their depressive status. The exhibition of immobility reflects the status of behavioral despair. In both genders, the duration of immobility was similar among genotypes (Figure 3A). We next placed mice on the elevated plus maze to assess the anxiety‐like behavior. The time spent in closed arms reflects the level of anxiety. Male Fb*Ccn2* ^−/−^ mice spent more time in the closed arms of the elevated plus maze than Fb*Ccn2* ^+/+^ mice (Figure 3B), indicating a higher anxiety level. However, in female mice, the time spent in the closed arms was comparable among genotypes. Taken together, mice lacking *Ccn2* exhibit normal locomotion and sensorimotor gating properties but anxiety‐like behavior in a sex‐dependent manner.

### 3.3 Elevated aggressive behaviors in Fb*Ccn2* ^−/−^ mice during RIT

**FIGURE 4:**
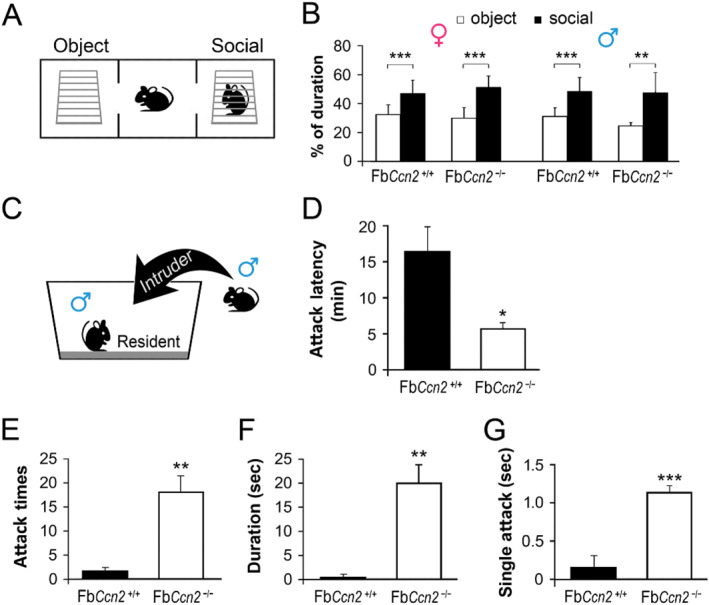
Social and aggressive behaviors in mice. Three‐chamber social interaction test was used to evaluate the sociability of mice (A). Fb*Ccn2* ^+/+^ and Fb*Ccn2* ^−/−^ mice of both genders spent a greater amount of time in the social chamber than in the object chamber (B). The aggressive behavior of male mice was evaluated using the resident‐intruder task (C). The latency of the first attack was shorter in Fb*Ccn2* ^−/−^ resident mice than in Fb*Ccn2* ^+/+^ mice (D), showing a sign of aggression. Further, the frequency of attacks (E) and the duration of the total and single attacks (F, G) also show greater aggression in Fb*Ccn2* ^−/−^ resident mice. *N* = 5 mice in each condition. Results are mean ± SEM. *p < 0.05; **p < 0.01; ***p < 0.001.

CCN2 is expressed in the OB.^9^, ^11^ Blocking of olfactory inputs or removal of the OB is known to influence emotion, sociability, and aggression.^32^, ^33^, ^34^ Given the removal of *Ccn2* influences the emotion in male mice, we wondered if sociability and aggression are also affected in these mutant mice. Three‐chamber social interaction test was used to evaluate the sociability of mice (Figure 4A). In both genders, all mice spent more time in the social chamber, where a target mouse was placed, than in the object chamber (Figure 4B), indicating the sociability of mice is not affected by the deletion of *Ccn2* in the forebrain.

The aggressive behavior was evaluated using the RIT. A male mouse was housed with a female mouse for 10 days as a resident. During the task, the female mouse was removed, and a male intruder mouse (wildtype) of matched size and weight was then introduced (Figure 4C). The reaction of the resident was measured. Compared with Fb*Ccn2* ^+/+^ mice, Fb*Ccn2* ^−/−^ mice exhibited elevated reactive aggression. In Fb*Ccn2* ^−/−^ resident mice, the latency to the first attack was shorter than that in Fb*Ccn2* ^+/+^ mice (Figure 4D), showing a sign of reactive aggression. Further, the frequency of attacks was higher (Figure 4E), and the duration of the total and single attacks was longer (Figure 4 F, G) in Fb*Ccn2* ^−/−^ resident mice. Together, we observed normal sociability but higher anxiety and aggression levels in male Fb*Ccn2* ^−/−^ mice.

### 3.4 Differential RIT‐induced c‐fos expression between Fb*Ccn2* ^+/+^ and Fb*Ccn2* ^−/−^ mice

**FIGURE 5:**
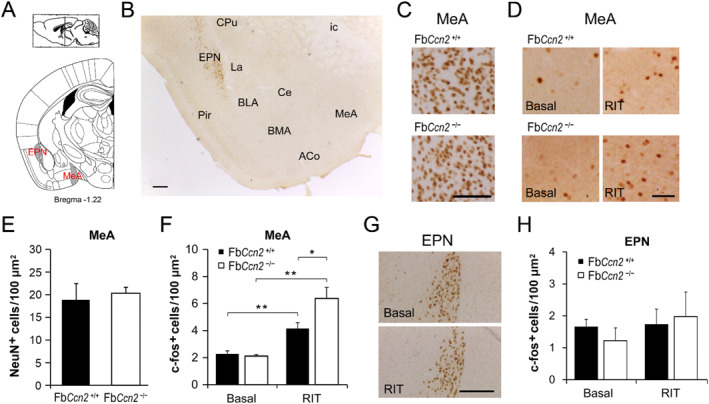
Intruder‐induced neuronal activity in the medial amygdala (MeA) and endopiriform nucleus (EPN). Schematic diagram of brain sections containing the MeA and EPN (A). Representative micrograph of CCN2 immunohistochemitry in Fb*Ccn2* ^+/+^ mice after RIT (B). CCN2‐positive cells are located in the EPN but not the amygdala (ACo, BLA, BMA, Ce, La, MeA). In the MeA, the density of NeuN‐positive cells was comparable between Fb*Ccn2* ^+/+^ and Fb*Ccn2* ^−/−^ mice (C, E). The basal c‐fos level was similar between genotypes, whereas the numbers of c‐fos‐positive cells were elevated after exposure to the intruder (RIT) in both genotypes. Notably, in the RIT group, the c‐fos level in Fb*Ccn2* ^−/−^ mice was higher than that in Fb*Ccn2* ^+/+^ mice (D, F). In the EPN, CCN2 expression was comparable between basal and RIT groups in Fb*Ccn2* ^+/+^ mice (G). RIT did not change the level of c‐fos‐positive cells in both Fb*Ccn2* ^+/+^ and Fb*Ccn2* ^−/−^ mice (H). *N* = 5 mice in each condition. Results are mean ± SEM. *p < 0.05; **p < 0.01. Scale bar = 200 μm in B and 100 μm in C and D. ACo, anterior cortical amygdaloid nucleus; BLA, basolateral amygdaloid nucleus; BMA, basomedial amygdaloid nucleus; Ce, central amygdaloid nucleus; CPu, caudate putamen; EPN, endopiriform nucleus; ic, internal capsule; La, lateral amygdaloid nucleus; Pir, piriform cortex; RIT, resident‐intruder task.

Some aggression‐related brain regions, such as the amygdala (AMY), are connected with the OB. In mice, CCN2 is not expressed in the AMY under normal conditions. We wondered if CCN2 expression could be induced by RIT. However, we did not observe CCN2 expression in the AMY of Fb*Ccn2* ^+/+^ mice following RIT (Figure 5B). Previous studies showed the index of neuronal activity, c‐fos expression, is elicited in the AMY following intermale fighting during the RIT.^39^, ^40^, ^41^ In the medial amygdala (MeA), the density of neurons (NeuN‐positive cells) was comparable between Fb*Ccn2* ^+/+^ and Fb*Ccn2* ^−/−^ mice (Figure 5C,E). The numbers of c‐fos‐positive cells were comparable between Fb*Ccn2* ^+/+^ and Fb*Ccn2* ^−/−^ mice without intruder exposure (as the basal level). In both genotypes, the numbers of c‐fos‐positive cells were elevated in resident mice after the RIT. Remarkably, in Fb*Ccn2* ^−/−^ mice, the c‐fos level was higher than that in Fb*Ccn2* ^+/+^ mice (Figure 5 D, F), in concert with the elevated reactive aggression observed in these mice. CCN2 is normally expressed in the endopiriform nucleus (EPN)^14^ (also see Figure 1 in the present study), which is also connected to the OB neurons. We wondered if its expression is changed following the exposure to the intruder. Compared with the basal level, the density of CCN2‐positive cells in the EPN was not altered after RIT (Figure 5G). The c‐fos level in the EPN was not changed after RIT in both Fb*Ccn2* ^+/+^ and Fb*Ccn2* ^−/−^ mice, either (Figure 5H).

**FIGURE 6:**
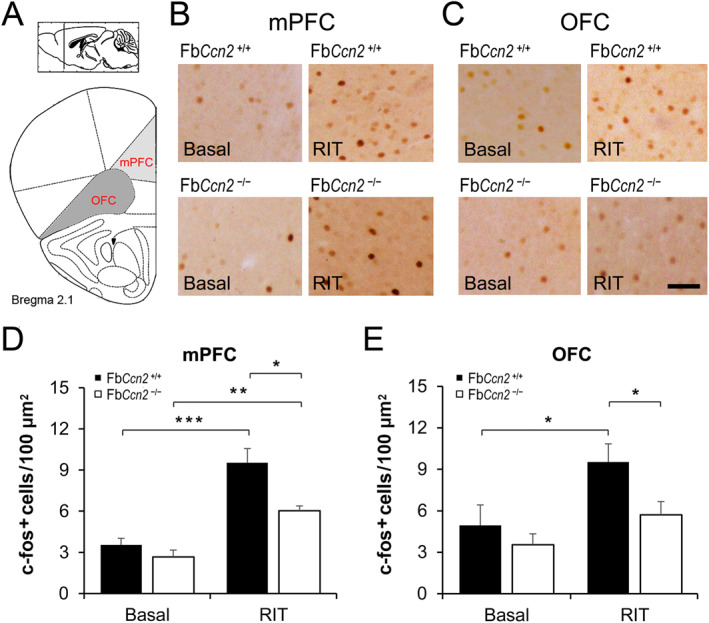
Intruder‐induced neuronal activity in the medial prefrontal cortex (mPFC) and orbitofrontal cortex (OFC). The numbers of c‐fos positive neurons were measured in brain sections containing the mPFC and OFC (A). In the mPFC, c‐fos positive neurons were comparable between genotypes in the basal condition and increased after RIT in both genotypes. Notably, in the RIT group, the c‐fos level in Fb*Ccn2* ^−/−^ mice was lower than that in Fb*Ccn2* ^+/+^ mice (B, D). In the OFC, the c‐fos expression was elevated after RIT in Fb*Ccn2* ^+/+^ mice but not in Fb*Ccn2* ^−/−^ mice (C, E). *N* = 5 mice in each condition. Results are mean ± SEM. RIT, resident‐intruder task. *p < 0.05; **p < 0.01; ***p < 0.001. Scale bar = 100 μm.

In the mouse brain, CCN2 is also expressed in the deep layer of mPFC and OFC (Figure 1B). These brain regions are highly associated with the inhibitory control of aggressive behaviors and emotional regulation.^29^, ^42^, ^43^ The density of CCN2‐positive cells in the mPFC and OFC was not affected by RIT. We then checked the c‐fos expression in the mPFC and OFC (Figure 6A). In the mPFC, the basal‐level c‐fos‐positive cells were comparable between Fb*Ccn2* ^+/+^ and Fb*Ccn2* ^−/−^ mice. After RIT, the numbers of c‐fos‐positive cells were elevated in mice of both genotypes; however, the c‐fos level in Fb*Ccn2* ^−/−^ mice was lower than that in Fb*Ccn2* ^+/+^ mice (Figure 6 B and D). In the OFC, compared with the basal level, the c‐fos expression was elevated in the RIT group in Fb*Ccn2* ^+/+^ mice; however, in intruder‐exposed Fb*Ccn2* ^−/−^ mice, the c‐fos expression was not significantly higher than those without intruder exposure (Figure 6C,E). These results suggested that in Fb*Ccn2* ^−/−^ mice, the mPFC and OFC neurons are not fully activated during RIT which may fail to suppress the aggression‐related neural circuit, resulting in their elevated aggressive behaviors in the RIT.

## 4 DISCUSSION

CCN2 is a secreted matricellular protein and may influence cell morphology and physiology in the brain in an autocrine and paracrine manner.^13^ CCN2 expression in the brain has been reported using CCN2‐EGFP mice. Based on the dendritic morphology, the GFP signals are expressed in neurons.^15^ In this study, we demonstrated that CCN2 is expressed in the forebrain, including the olfaction‐related regions such as the glomerular layer of the OB, AON, and EPN as well as the deep portion of the mPFC, OFC, and cortical layer VIb (Figure 1). Most of the regions are involved in the olfactory function and emotional control. We showed for the first time that male mice lacking the *Ccn2* gene in the forebrain exhibit signs of anxiety (Figure 3) and elevated reactive aggression (Figure 4) but normal locomotion, sensorimotor gating, and social behaviors (Figure 2).

The most significant phenotype of Fb*Ccn*2KO mice is elevated reactive aggression, which is highly associated with olfactory perception and emotional control. Increased neuronal activity in the MeA is closely related to greater attack responses.^44^, ^45^ The RIT‐induced c‐fos pattern showed hyperactivity in the MeA of Fb*Ccn*2KO mice (Figure 5) explaining the aggressive phenotype. On the other hand, in the region for suppression of aggression and regulation of emotion, the prefrontal cortex (mPFC and OFC), RIT‐induced c‐fos expression in Fb*Ccn*2KO mice was less active than WT controls (Figure 6) which is in line with the elevated reactive aggression behavior in KO mice. Based on these findings, we proposed that loss of CCN2 in the forebrain, especially the olfaction‐ and emotion‐related regions might alter the perception of olfactory social signals and inhibitory control of aggression that exhibit greater reactive aggression in the presence of an unfamiliar intruder.

Using an RNAi‐mediated strategy, *Ccn2* has been locally knocked down in the OB since the neonatal era. In this *Ccn2* knockdown model, the survival of periglomerular inhibitory neurons, activity of mitral cells as well as olfactory behaviors are affected. These *Ccn2* knockdown mice exhibited better odorant detection and olfactory discrimination.^11^ In our model, the expression of *Emx1* starts as early as embryonic day 10.5^35^; it is believed that forebrain‐specific removal of *Ccn2* is executed during the embryonic period. Along with the findings in the *Ccn2* knockdown model,^11^ we speculated an increased sensitivity of olfactory social threats in our Fb*Ccn2* KO mice during the exposure of an intruder.

The glomeruli of the main OB receive odor signals from the olfactory epithelium and innervate the olfactory brain areas, including the AON, piriform cortex, EPN, the lateral entorhinal cortex, and the MeA.^46^, ^47^ Further, MeA projects to the thalamus and various nuclei in the hypothalamus. Thalamic relay nuclei forward olfaction‐related information to the mPFC. It is proposed that the aggression‐provoking olfactory cues are detected by the olfactory systems and processed in the core aggression circuit.^31^ The activity of the aggression circuit, which consists of several interconnected nuclei, including the MeA, the bed nucleus of stria terminalis, and some hypothalamic nuclei, such as the paraventricular nucleus of the hypothalamus, could determine the degree of aggressive arousal and the potential of attack.^31^, ^48^ In male Fb*Ccn2* ^−/−^ mice, the perception of social olfactory cues might be altered and misinterpreted as strong aggression‐provoking social threat signals and resulted in increased MeA neuronal activity (Figure 5 D, F) and greater aggressive behaviors (Figure 4).

Aggression is thought to be an instinctual behavior.^49^ Without exhibiting aggression, one could not protect or defend in response to an imperative threat. However, it is important to decide when to display aggression and when to suppress aggressive impulses. In fact, the ability to suppress aggressive behaviors develops with the maturation of the brain.^50^ The prefrontal cortex, one of the last regions to reach maturity in the brain, plays a critical role in the modulation of aggression.^51^, ^52^ Increased aggressiveness has been noted in rats with OFC damage, indicating that the OFC may serve an inhibitory role in aggressive behavior.^53^ Using an optogenetic approach, activation of excitatory neurons in the mPFC inhibits inter‐male aggression in mice.^54^ Topiramate, an antiepileptic drug, suppresses isolation‐induced aggression in mice.^41^ In isolated mice treated with Topiramate, the c‐fos expression is increased in the OFC and mPFC but decreased in the MeA compared with those without Topiramate treatment. These results indicate an inverted relationship between the prefrontal cortex activity and aggressive behaviors in rodents. We thus suggested that the activity of projecting neurons in the prefrontal cortex could inhibit the activation of MeA and thus suppress aggressive behavior.

In male Fb*Ccn2* ^−/−^ mice, the c‐fos expression in the mPFC was elevated after RIT, but the level was significantly lower than that in Fb*Ccn2* ^+/+^ mice (Figure 6 B and D), indicating a weaker cortical suppressive activity for aggression in Fb*Ccn2* ^−/−^ mice. The findings in the OFC further supported this notion. In Fb*Ccn2* ^+/+^ mice, the c‐fos expression was increased after RIT, while in Fb*Ccn2* ^−/−^ mice, the c‐fos expression of the RIT group was comparable to the basal level (Figure 6 C and E), again indicating a weaker cortical inhibitory signal in the OFC. We, therefore, speculated that during the RIT, the cortical neurons in the mPFC and OFC of Fb*Ccn2* ^−/−^ mice are not fully activated and then fail to suppress the aggression‐related neural circuit. Since CCN2 is expressed in the deep layers of the mPFC and OFC, it may modulate the activity of nearby cortical neurons in a paracrine manner. In our previous study, the results suggested that CCN2 in the cortical layer VIb might regulate the maturation of nearby oligodendrocytes in a paracrine manner.^13^ Alternatively, a lack of CCN2 may affect the properties of projecting cortical neurons during development. An inducible forebrain‐specific *Ccn2*KO model could resolve this issue.

The prefrontal cortex plays an important role in emotional control.^52^ CCN2 expression in the mPFC and OFC is absent in male Fb*Ccn2* ^−/−^ mice which display signs of anxiety. The release of CCN2 in the prefrontal cortex might mediate emotional functions in male mice. The link between prefrontal CCN2 expression and emotional regulation is thus suggested. An earlier study reported that intracerebroventricular administration of an anti‐CCN2 antibody (FG‐3019) could decrease depression‐like behavior and suggested CCN2 as a pro‐depressant.^25^ It would be interesting to test if an injection of FG‐3019 affects social and aggressive behaviors.

In the nervous system, the expression of CCN2 can be induced under pathological or stressful conditions.^12^, ^16^, ^17^, ^18^, ^19^, ^20^, ^21^, ^22^, ^23^, ^24^, ^25^, ^26^, ^55^ However, in our model, CCN2 in the brain was not immediately elevated after the encounter with the intruder. We might evaluate the role of stress‐induced CCN2 expression in the forebrain by adopting the repeated social defeat paradigm.^25^, ^26^ Besides, in our future study, we need to explore aggressive behaviors in female mice. The function of CCN2 in the brain will be elucidated in a sex‐dependent manner.

CCN3, similar to CCN2, belongs to the CCN family.^56^, ^57^ CCN3 plays an opposing role to CCN2, creating a Yin‐Yang collaborative relationship.^57^, ^58^ In the cartilage, CCN2 and CCN3 play vital roles in chondrocyte differentiation in a cooperative way^59^, ^60^; while in the kidney, CCN2 promotes cell proliferation, yet CCN3 inhibits it.^61^, ^62^ CCN2 inhibits the maturation of oligodendrocytes,^10^ while regulatory T cell‐derived CCN3 enhances the differentiation of oligodendrocyte progenitor cells.^63^ In our Fb*Ccn2* ^−/−^ mice, increased mature oligodendrocytes are noticed in the external capsule,^13^ supporting the suppressive effect of CCN2 on oligodendrocytes. In CCN3 knockout mice^64^, the number of oligodendrocytes is comparable to wildtype control mice in healthy or demyelinated conditions, suggesting that CCN3 is not essential in myelination or remyelination.^65^ It has been observed that the overexpression of CCN3 leads to the inhibition of axonal projection.^66^ Both CCN2 and CCN3 are expressed in the brain,^8^, ^9^, ^67^ including the OB and olfactory peduncle.^68^ The knockout of *Ccn2* in the forebrain may have an impact on CCN3 expression in the same forebrain structures. This alteration in CCN3 levels may influence the regulation and projection of neurons, ultimately resulting in abnormal behaviors. Further exploration of CCN3 expression in the Fb*Ccn2* ^*−/−*^ mice is warranted.

Our study characterized for the first time that mice lacking CCN2 in the forebrain display signs of anxiety and elevated reactive aggression. The role of CCN2 in brain function is demonstrated. Subjects with neuropsychiatric disorders suffering anxiety‐ or depression‐related symptoms sometimes exhibit excessive aggression.^69^, ^70^ Our animal model would be useful in elaborating the mechanism underlying anxiety and reactive aggressive behaviors and the development of therapeutic strategies.

## AUTHOR CONTRIBUTIONS

Conceptualization: Li‐Jen Lee, Kuang‐Yung Lee; Data curation: Ho‐Ching Chang and Li‐Jen Lee; Writing original draft: Ho‐Ching Chang, Chi‐Hou Ng, Li‐Jen Lee, Kuang‐Yung Lee; Investigation: Ho‐Ching Chang, Yu‐Fu Chen, Yu‐Chun Wang, I‐Shing Yu, Lukas Jyuhn‐Hsiarn Lee, Li‐Jen Lee, Kuang‐Yung Lee; Project Administration: Kuang‐Yung Lee. All authors have read and approved the submitted version of the manuscript.

## CONFLICT OF INTEREST STATEMENT

No conflicts of interest were reported by the authors.

## ETHICS STATEMENT

Mice were used in this study. All animal procedures were approved by the Institutional Animal Care and Use Committee of the College of Medicine, National Taiwan University (approval code: 20170291).

## Data Availability

The authors confirm that the data supporting the findings of this study are available within the article.
